# Metabolic Engineering of *Saccharomyces cerevisiae* for Enhanced Dihydroartemisinic Acid Production

**DOI:** 10.3389/fbioe.2020.00152

**Published:** 2020-03-17

**Authors:** Bo-Xuan Zeng, Ming-Dong Yao, Ying Wang, Wen-Hai Xiao, Ying-Jin Yuan

**Affiliations:** ^1^Frontier Science Center for Synthetic Biology and Key Laboratory of Systems Bioengineering (Ministry of Education), School of Chemical Engineering and Technology, Tianjin University, Tianjin, China; ^2^Collaborative Innovation Center of Chemical Science and Engineering (Tianjin), Tianjin University, Tianjin, China

**Keywords:** dihydroartemisinic acid, artemisinic acid, protein engineering, synthetic biology, *Saccharomyces cerevisiae*

## Abstract

Direct bioproduction of DHAA (dihydroartemisinic acid) rather than AA (artemisinic acid), as suggested by previous work would decrease the cost of semi-biosynthesis artemisinin by eliminating the step of initial hydrogenation of AA. The major challenge in microbial production of DHAA is how to efficiently manipulate consecutive key enzymes ADH1 (artemisinic alcohol dehydrogenase), DBR2 [artemisinic aldehyde Δ11(13) reductase] and ALDH1 (aldehyde dehydrogenase) to redirect metabolic flux and elevate the ratio of DHAA to AA (artemisinic acid). Herein, DHAA biosynthesis was achieved in *Saccharomyces cerevisiae* by introducing a series of heterologous enzymes: ADS (amorpha-4,11-diene synthase), CYP71AV1 (amorphadiene oxidase), ADH1, DBR2 and ALDH1, obtaining initial DHAA/AA ratio at 2.53. The flux toward DHAA was enhanced by pairing fusion proteins DBR2-ADH1 and DBR2-ALDH1, leading to 1.75-fold increase in DHAA/AA ratio (to 6.97). Moreover, to promote the substrate preference of ALDH1 to dihydroartemisinic aldehyde (the intermediate for DHAA synthesis) over artemisinic aldehyde (the intermediate for AA synthesis), two rational engineering strategies, including downsizing the active pocket and enhancing the stability of enzyme/cofactor complex, were proposed to engineer ALDH1. It was found that the mutant H194R, which showed better stability of the enzyme/NAD^+^ complex, obtained the highest DHAA to AA ratio at 3.73 among all the mutations. Then the mutant H194R was incorporated into above rebuilt fusion proteins, resulting in the highest ratio of DHAA to AA (10.05). Subsequently, the highest DHAA reported titer of 1.70 g/L (DHAA/AA ratio of 9.84) was achieved through 5 L bioreactor fermentation. The study highlights the synergy of metabolic engineering and protein engineering in metabolic flux redirection to get the most efficient product to the chemical process, and simplified downstream conversion process.

## Introduction

Artemisinin, a well-known sesquiterpene lactone isolated from extracts of *Artemisia annua* with excellent anti-malaria properties, had been designated as first-line antimalarial drugs by WHO in 2002 (Paddon and Keasling, 2014). Artemisinin production and prices vary greatly as they depend on plant extraction, which relies on the supply of plant materials (Chunyin and Cook, 2012; Peplow, 2013). Therefore, a stable and sustainable supply of artemisinin is highly desirable. This breakthrough was achieved by the Amyris, Inc., in which a semi-synthetic process of artemisinin production was developed (Westfall et al., 2012; Paddon et al., 2013). The semi-synthesis of artemisinin consists of two parts: (1) *de novo* biosynthesis of AA (artemisinic acid) with a very high titer (25 g/L) by an engineered *Saccharomyces cerevisiae* and (2) extract AA from yeast culture and transform it to artemisinin by chemical process. To be noted, the first step of the chemical process was reduction of AA to dihydroartemisinic acid (DHAA). Although the biosynthesis of DHAA has been demonstrated (Zhang et al., 2008), the productivity (about 15.7 mg/L) has not yet to be optimized in a heterologous host. Although hydrogenation conversion of AA to DHAA was efficient (conversion efficiency 99%), the production of unexpected isoform (R, S)-DHAA (about 6%) was not avoided (Paddon et al., 2013; Turconi et al., 2014; Pieber et al., 2015). Instead, biosynthesis of DHAA produced very little (R, S) isomer in yeast (Zhang et al., 2008). In order to execute a more efficient way of synthesizing artemisinin, engineering DHAA biosynthesis in microbes would open up a promising alternative route.

**FIGURE 1:**
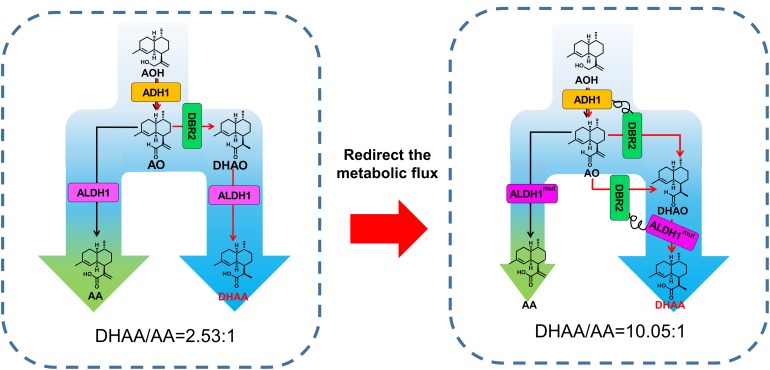
Schematic diagram of tailoring the ratio of DHAA to AA by protein fusion and modifying ALDH1 catalysis preference. The broader blue arrow indicates enhanced flux toward DHAA. AOH, artemisinic alcohol; AO, artemisinic aldehyde; DHAO, dihydroartemisinic aldehyde; ADH1, artemisinic alcohol dehydrogenase of *A. annua*; DBR2, artemisinic aldehyde Δ11(13) reductase of *Artemisia annua*; ALDH1, artemisinic aldehyde dehydrogenase of *A. annua.*

The biosynthesis pathways of DHAA and AA both start from amorpha-4,11-diene that is oxidized to AO (artemisinic aldehyde) through AOH (artemisinic alcohol) by CYP17AV1 (amorphadiene oxidase) and ADH1 (artemisinic alcohol dehydrogenase) (Figure 1). AO is a joint intermediate followed by two branch biosynthesis pathways: (1) directly oxidized to AA by ALDH1 (aldehyde dehydrogenase) or (2) reduced to dihydroartemisinic aldehyde (DHAO) by DBR2 (artemisinic aldehyde Δ11(13) reductase) and then oxidized to DHAA by ALDH1 (Chen et al., 2017). The enzymatic parameters of DBR2 and ALDH1 to their substrates AO and DHAO were summarized in Supplementary Table S1 (Zhang et al., 2008; Teoh et al., 2009). Because of the slightly higher reported affinity of ALDH1 [For substrate AO, Km(DBR2) = 19 μM, Km(ALDH1) = 2.58 μM], there is no advantage for DBR2 to bind with AO. Furthermore, no enzymes (even DBR2) have been reported to directly catalyze AA to DHAA. Thus, it was inevitable to produce a large amount of AA as by-product of DHAA production. So far, the highest ratio of DHAA/AA reaches just 1.67 achieved by enzymatic reaction *in vitro* (Chen et al., 2017). Therefore, the major challenge in production of DHAA by recombinant yeasts is efficient redirection of carbon flux to DHAA rather than AA.

Branch-point regulatory mechanisms are involved in many natural metabolic pathways (such as TCA cycle) (Chen et al., 2017). In these pathways, the enzymes can work together spatiotemporally due to protein–protein interactions and channel the metabolites between sequential enzymes without equilibration in the aqueous phase inside cells (Sweetlove and Fernie, 2018). The assemblies of consecutive enzymes are formed either from large clusters of multiple copies of enzymes, or by pairwise interactions of enzymes from single complexes which are beneficial for enzymes to reach substrate saturation (Zhang, 2011) so that the reaction flux is regulated at a branch point. Pham et al. (2015) once co-localized enzymes responsible for GABA (Gamma-aminobutyric acid) biosynthesis together to switch the metabolic flux toward GABA from TCA cycle and finally increased the production of GABA by 2.7 fold. Referring to DHAA biosynthetic pathway itself and key enzymes, two obstacles should be overcome to achieve a high ratio of DHAA/AA and a high DHAA titer: (1) redirecting the metabolic flux from AA toward DHAA *via* the expression of DBR2 requires the assembly of the pathway enzyme in a desired order and promotes the reactions of metabolites along a specified pathway (Miles et al., 1999); (2) rationally engineering ALDH1 to shift the substrate specificity from AO to DHAO.

Herein, the biosynthesis pathway of DHAA was successfully rebuilt in *S. cerevisiae* with high FPP supply. In order to improve the ratio of DHAA/AA by increasing the substrate accessibility of AO by DBR2 as well as that of DHAO by ALDH1, fusion proteins of paired enzymes (ADH1-DBR2 or DBR2-ALDH1) were adopted to reorganize the biosynthetic pathway of DHAA (Figure 1). Meanwhile, ALDH1, as the joint enzyme for biosynthesis of DHAA and AA, was also rationally engineered to shift the substrate specificity from AO to DHAO. Correspondingly, the ratio of DHAA/AA was enhanced by 3.34 fold (from 2.53 to 10.05) through integrating these above two strategies, without the compromise of DHAA production. Eventually, the highest DHAA titer of 1.70 g/L (DHAA/AA ratio of 9.84) was achieved in a 5 L bioreactor through high density fermentation. The study highlights the importance of redirecting metabolic flux toward a desired target *via* consecutive enzyme-enabled reorganization.

## Materials and Methods

### Strains and Medium

All *E. coli* used for plasmid construction were cultured at 37°C in Luria-Bertani (LB) medium (1% tryptone, 0.5% yeast extract, and 1% NaCl) with 100 μg/ml ampicillin or 34 μg/ml chloramphenicol if necessary.

All engineered yeast strains were derived from *S. cerevisiae* CEN.PK2-1C (Entian and Kötter, 2007) obtained from EUROSCARF (Frankfurt, Germany) and were listed in Table 1. *S. cerevisiae* strains were cultured in YPD medium (2% tryptone, 1% yeast extract, and 2% glucose) or in synthetic complete (SC) drop-out medium at 30°C. All the medium formulations for yeast culture are available in our previous work (Su et al., 2015).

**TABLE 1:** Yeast strains used in this study.

Yeast strains	Description	Source
CEN.PK2.1C	*MAT a; ura3-52, trp1-289, leu2-3,112, his3*Δ*1, MAL2-8C, SUC2*	Invitrogen
Sc027	CEN.PK2-1C derivative*; leu2-3,112:G418^*R*^_*P*_*GAL*__7_-CYB5_T_*ERG*__19_(RC)-ERG19(RC)-P_*GAL*__1_(RC)_P_*GAL*__10_-ERG8-T_*ERG*__8_; his3*Δ*1:HIS3_P_*GAL*__7_-ALDH1-T_*TDH*__1__*T*_*ERG*__12_(RC)-ERG12(RC)-P_*GAL*__1_(RC)_P_*GAL*__10_-ERG10-T_*ERG*__10_*; *ade1*Δ*:T_*HMG*__1_(RC)-tHMG1(RC)-P_*GAL*__1_(RC)_P_*GAL*__10_-IDI1-T_*IDI*__1__ADE1; ura3-52:T_*HMG*__1_(RC)-tHMG1(RC)-P_*GAL*__1_(RC)_P_*GAL*__10_-ERG13-T_*ERG*__13_; trp1-289:T_*HMG*__1_(RC)-tHMG1(RC)-P_*GAL*__1_(RC)_P_*GAL*__10_-ERG20-T_*ERG*__20__TRP1; gal1/10/7*Δ*:natA_P_*GAL*__3_-CPR1-T_*CYC*__1_*;	This study
Sc057	Sc027 derivative*; GAL80*Δ*:URA3_P_*GAL*__7_-AaADH1-T_*TDH*__1_*	This study
Sc077	Sc027 derivative; *GAL80*Δ*:URA3*	This study
Sc085	Sc057 derivative; pZBX040	This study
Sc113	Sc077 derivative; pZBX059	This study
Sc115	Sc077 derivative; pZBX067	This study
Sc146	Sc077 derivative; pZBX060	This study
Sc147	Sc077 derivative; pZBX069	This study
Sc352	Sc057 derivative; *his3*Δ*aldh1*Δ*:hphA*	This study
Sc361	Sc077 derivative; pZBX067; Δ*ALDH1:P_*GAL*__7_-DBR2-Linker1-ALDH1-T_*TDH*__1_*	This study
Sc467	Sc057 derivative; pZBX040*;* Δ*ALDH1:P_*GAL*__7_-DBR2-Linker2-ALDH1-T_*TDH*__1_*	This study
Sc468	Sc057 derivative; pZBX040*;* Δ*ALDH1:P_*GAL*__7_-DBR2-Linker1-ALDH1-T_*TDH*__1_*	This study
Sc470	Sc085 derivative; *ura3down:hphMX6-P_*GAL*__7_-DBR2-T_*CYC*__1_*	This study
Sc429	Sc057 derivative; pZBX040 Δ*ALDH1:P_*GAL*__7_-ALDH1^*H*194*R*^-T_*TDH*__1_*	This study
Sc457	Sc057 derivative; pZBX067; Δ*ALDH1:P_*GAL*__7_-DBR2-Linker1-ALDH1^*H*194*R*^-T_*TDH*__1_*	This study

*The DNA fragment followed by ‘(RC)’ represents that the orientation of the DNA fragment is reversed. Sequences of linker1 and linker2 showed in Table 3; In strain Sc470, the cassette hphMX6-P_*GAL*__7_-DBR2-T_*CYC*__1_ was inserted into the downstream of cassette *T_*HMG*__1_(RC)-tHMG1(RC)-P_*GAL*__1_(RC)_P_*GAL*__10_-ERG13-T_*ERG*__13_* which had been integrated in the locus in *ura3.**

### Plasmid Construction

Plasmids used in this study are listed in Table 2. The genes *ADS* (ACCESSION Q9AR04), *CYP71AV1* (ACCESSION Q1PS23), *DBR2* (ACCESSION KC505370.1), *ALDH1* (ACCESSION JQ609276.1), *ADH1* (ACCESSION JF910157.1), *CYB5* (ACCESSION JQ582841.1), *CPR1* (ACCESSION DQ318192.1) from *Artemisia annua* were codon-optimized for expression in yeast and synthesized by GenScript, Inc. (China). To overexpress three key genes for DHAA biosynthesis (including ADS, CYP71AV1, and DBR2), plasmid pZBX040 were first constructed based on the multi-copy plasmid pRS425. Then the plasmid with fusion protein DBR2-ADH1 or ADH1-DBR2 (including pZBX059, pZBX060, pZBX067, pZBX069) were constructed based on pZBX040. To substitute ALDH1 integrated in the genome with fusion protein DBR2-ALDH1, the cassette *HIS3-T_*HIS*__3_-P_*GAL*__7_-DBR2-Linker-ALDH1-T_*TDH*__1_* were assembled in pSB1C3 (obtained from the Registry of Standard Biological Parts^1^) to form plasmid pZBX100, pZBX101. The cassettes with mutated ALDH1 were constructed based on pZBX101. All the primers used in this work are listed in Supplementary Table S2.

**TABLE 2:** Plasmids used in this study.

Plasmid	Description	Source
pZBX020	*pRS425_T_*ADH*__1_(RC)-CYP71AV1(RC)-P_*GAL*__10_(RC)_P_*GAL*__1_-ADS-T_*PGK*__1_*	This study
pZBX040	*pRS425_P_*GAL*__7_-DBR2-T_*CYC*__1__*T*_*ADH*__1_(RC)-CYP71AV1(RC)-P_*GAL*__10_(RC)_P_*GAL*__1_-ADS-T_*PGK*__1_*	This study
pZBX059	*pRS425_P_*GAL*__7_-ADH1-linker1-DBR2-T_*CYC*__1__*T*_*ADH*__1_(RC)-CYP71AV1(RC)-P_*GAL*__10_(RC)_P_*GAL*__1_-ADS-T_*PGK*__1_*	This study
pZBX060	*pRS425_P_*GAL*__7_-ADH1-linker2-DBR2-T_*CYC*__1__*T*_*ADH*__1_(RC)-CYP71AV1(RC)-P_*GAL*__10_(RC)_P_*GAL*__1_-ADS-T_*PGK*__1_*	This study
pZBX067	*pRS425_P_*GAL*__7_-DBR2-linker1-ADH1-T_*CYC*__1__*T*_*ADH*__1_(RC)-CYP71AV1(RC)-P_*GAL*__10_(RC)_P_*GAL*__1_-ADS-T_*PGK*__1_*	This study
pZBX069	*pRS425_P_*GAL*__7_-DBR2-linker2-ADH1-T_*CYC*__1__*T*_*ADH*__1_(RC)-CYP71AV1(RC)-P_*GAL*__10_(RC)_P_*GAL*__1_-ADS-T_*PGK*__1_*	This study
pZBX101	*pSB1C3_HIS3-T_*HIS*__3__*P*_*GAL*__7_-DBR2-Linker1-ALDH1-T_*TDH*__1_*	This study
pZBX100	*pSB1C3_HIS3-T_*HIS*__3__*P*_*GAL*__7_-DBR2-Linker2-ALDH1-T_*TDH*__1_*	This study
pZBX199	*pSB1C3_HIS3-T_*HIS*__3__*P*_*GAL*__7_-ALDH1^*H*194*R*^-T_*TDH*__1_*	This study
pZBX196	*pSB1C3_HIS3-T_*HIS*__3__*P*_*GAL*__7_-ALDH1^*G*227*V*^-T_*TDH*__1_*	This study
pZBX197	*pSB1C3_HIS3-T_*HIS*__3__*P*_*GAL*__7_-ALDH1^*G*227*F*^-T_*TDH*__1_*	This study
pZBX194	*pSB1C3_HIS3-T_*HIS*__3__*P*_*GAL*__7_-ALDH1^*G*223*V*^-T_*TDH*__1_*	This study
pZBX195	*pSB1C3_HIS3-T_*HIS*__3__*P*_*GAL*__7_-ALDH1^*G*223*F*^-T_*TDH*__1_*	This study
pZBX218	*pSB1C3_HIS3-T_*HIS*__3__*P*_*GAL*__7_-DBR2-Linker1-ALDH1^*H*194*R*^-T_*TDH*__1_*	This study

#### Construction of pZBX040

The DNA fragments *ADS*, *CYP71AV1*, *P_*GAL*__1_,_10_*, *T_*PGK*__1_*, and *T_*ADH*__1_* were amplified by PCR and joined together by overlap extension PCR(OE-PCR) to obtain cassette *T_*ADH*__1_(RC)-CYP71AV1(RC)-P_*GAL*__10_(RC)_P_*GAL*__1_-ADS-T_*PGK*__1_.* The cassette was digested with *Bam*HI and *Xho*I and inserted into pRS425 to obtain plasmid pZBX020. Fragments *DBR2*, *P_*GAL*__7_*, *T_*CYC*__1_* were amplified by PCR and joined together by OE-PCR to obtain cassette *P_*GAL*__7_-DBR2-T_*CYC*__1_*. The cassette was digested with *Xho*I and *Pst*I and inserted into pZBX020 to obtain plasmid pZBX040.

#### Construction of Plasmids for Fusion Protein ADH1-DBR2 and DBR2- ADH1

Fragments *P_*GAL*__7_-ADH1, DBR2-T_*CYC*__1_* were amplified by PCR and then assembled together to obtain cassettes of different types of *P_*GAL*__7_-ADH1-linker-DBR2-T_*CYC*__1_* by OE-PCR. These cassettes were digested with *Xho*I and *Pst*I and inserted into pZBX020 to obtain the plasmids with the fusion protein *ADH1-DBR2* (including pZBX059, pZBX060). Fragments *P_*GAL*__7_-DBR2, ADH1, T_*CYC*__1_* were amplified by PCR and then linked together to form cassettes of different types of *P_*GAL*__7_-DBR2-linker-ADH1-T_*CYC*__1_* by OE-PCR. These cassettes were digested with *Xho*I and *Pst*I and inserted into pZBX020 to obtain the plasmids with the fusion protein *DBR2-ADH1* (including pZBX067, pZBX069). The sequences of two linkers in the fusion proteins used in this work are listed in Table 3.

**TABLE 3:** Linkers used in this study.

Linker	Sequence	Type
Linker1	EAAAKEAAAKA	Rigid
Linker2	GGGGSGGGGSGGGGS	Flexible

#### Construction of Plasmid for Fusion Protein DBR2-ALDH1

Fragment *HIS3-P_*GAL*__7_* was amplified by PCR from the genome of Sc085 followed by digestion of *Eco*RI and *Bsa*I. The fragments of *DBR2*, and *ALDH1-T_*TDH*__1_* were amplified by PCR from Sc085. Fragment *DBR2* was digested with *Bsa*I, and fragment *ALDH1-T_*TDH*__1_* was digested with *Bsa*I and *Pst*I. The vector pSB1C3 was digested with *Eco*RI, and *Bsa*I. All above four fragments were ligated together by T4 ligase to construct all the plasmid with the fusion protein *DBR2-ALDH1* (including pZBX099, pZBX100, pZBX101). The cassettes of the plasmids were released from plasmid followed by digestion of *Pme*I before transformation to the strain Sc352 for integration.

#### Construction of Plasmid for ALDH1 Mutants

Each DNA fragment of an ALDH1 mutant was divided into two parts *P_*GAL*__7_-ALDH1a* and *ALDH1b* with 40 bp overlapping according to the mutated site, and was amplified by PCR. Each pair of *P_*GAL*__7_-ALDH1a* and *ALDH1b* was ligated to form intact ALDH1 mutant by OE-PCR followed by digestion of *Spe*I and *Kpn*I. The vector pZBX100 was digested with *Spe*I, and *Kpn*I for insertion of the fragment of P_*GAL*__7_-ALDH1 mutant, obtaining pZBX194, pZBX195, pZBX196, pZBX197, and pZBX199.

The process of construction of pZBX218 was the same as that of pZXB101 except that the fragment *ALDH1-T_*TDH*__1_* was amplified from plasmid pZBX199. All the cassettes of the plasmid described here were released from plasmid by digestion of *Pme*I and transformed to the strain Sc352.

### Fermentation Condition

#### Medium for Fermentation

All the fermentation medium (FM) recipe used in this work was prepared similarly to that used in Westfall’s work with some modifications (Westfall et al., 2012). The medium was composed of 8 g/L KH_2_PO_4_, 15 g/L (NH4)_2_SO_4_, 6.2 g/L MgSO_4_⋅7H_2_O, 40 g/L glucose, 12 ml/L vitamin solution, and 10 ml/L trace metal solution. The vitamin solution included 0.05 g/L biotin, 1 g/L calcium pantothenate,1 g/L nicotinic acid, 25 g/L myo-inositol,1 g/L thiamine HCl, 1 g/L pyridoxal HCl, 0.2 g/L *p*-aminobenzoic acid and 2 g/L adenine sulfate. The trace metal solution was composed of 5.75 g/L ZnSO_4_⋅7H_2_O, 0.32 g/L MnCl_2_⋅4H_2_O, 0.32 g/L Anhydrous CuSO_4_, 0.47 g/L CoCl_2_⋅6H_2_O, 0.48 g/L Na_2_MoO_4_⋅2H_2_O, 2.9 g/L CaCl_2_⋅2H_2_O, 2.8 g/L FeSO_4_⋅7H_2_O, and 80 ml/L EDTA solution (containing 0.5 mol/L Na_2_EDTA pH = 8.0). The amino acid solution (10 ml/L), including 2 g/L methionine, 6 g/L tryptophan, 8 g/L isoleucine, 5 g/L phenylalanine, 10 g/L sodium glutamate, 20 g/L threonine, 10 g/L aspartate, 15 g/L valine, 40 g/L serine and 2 g/L arginine, was supplemented to the medium in our study for obtaining better cell growth use. The FM bases (8 g/L KH_2_PO_4_, 15 g/L (NH_4_)_2_SO_4_, and 6.2 g/L MgSO_4_⋅7H_2_O) and glucose stock solution (667 g/L) were sterilized using an autoclave. Vitamin solution, trace metal solution, and amino acid solution were sterilized by filtration. All the components of the FM were mixed together after sterilization. Glucose stock solution (667 g/L), filtrated 95% (v/v) ethanol solution and filtrated feed stock solution (80 g/L KH_2_PO_4_, 150 g/L (NH4)_2_SO_4_, 62 g/L MgSO_4_⋅7H_2_O) also needed to be prepared for 5 L bioreactor fermentation. pH was adjusted to 5 by 10 mol/L NaOH prior to use.

#### Flask Fermentation

To prepare seed vials, single isolates of each strain from agar plates were grown for 18 h in FM medium at 30°C and 200 rpm. And then the cultures were inoculated into fresh FM medium at initial OD_600_ of 0.05 and grown for another 16 h cultivation at 30°C. The seed culture was transferred into 250 ml flask containing 25 ml FM medium at initial OD_600_ of 0.2. The cells were grown at 30°C with shaking at 200 rpm. After 24 h, 5 ml IPM (Isopropyl myristate) and 20 g/L ethanol was added to each flask. The whole fermentation process continued for 120 h until harvest.

#### 5 L Bioreactor Fermentation

Seed culture preparation was the same as in-flask fermentation. The seed culture (200 mL) was inoculated into a 5 L bioreactor containing a 2 L batch FM medium with 20 g/L glucose. The pH was controlled at 5 by adding 5 mol/L NaOH. The gas flow and the temperature were maintained at 1.5 vvm and 30°C. During the fermentation, glucose solution was fed into the bioreactor at a speed of 0.3 mL/min after the glucose was depleted at the running time of about 6 h. During the glucose feeding stage, the dissolved oxygen level (DO) was kept at 40% by cascading agitation from 400 to 600 rpm. When OD_600_ increased to 50 (about 30–36 h), the feeding of glucose was switched to ethanol feeding. The concentration of ethanol was maintained at 10 g/L to guarantee no starvation and no excess carbon accumulation. During the ethanol feeding process, DO was automatically maintained at about 30% by cascading stirring.

### The Measurement of DHAA and AA and Other Intermediates

After harvest, the fermentation broth was centrifuged at 12,000 *g* for 2 min and the IPM phase was collected. And then 50 μL organic phase was mixed with 950 μL methanol. After filtrated with 0.22 μm Nylon66 filter, the sample was ready for HPLC analysis.

**FIGURE 2:**
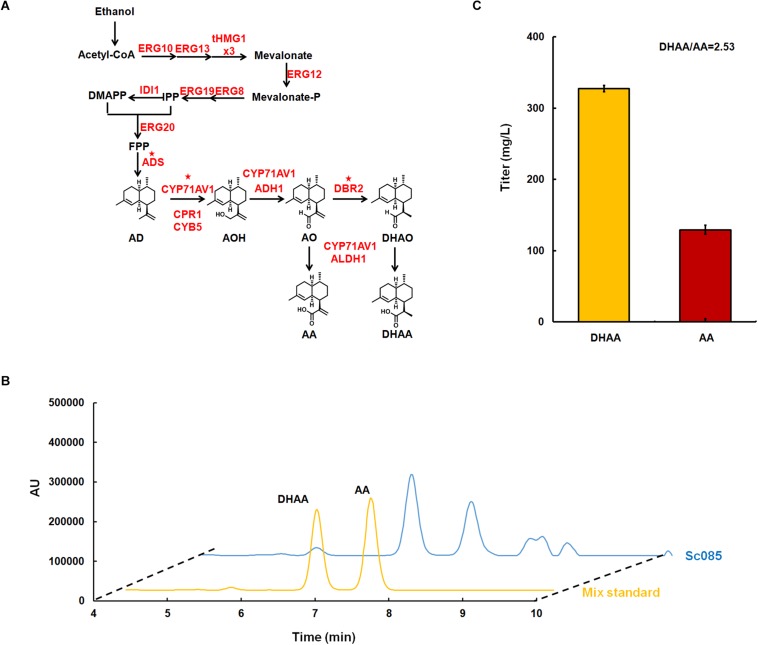
Microbial production of dihydroartemisinic aldehyde in *Saccharomyces. cerevisiae*. **(A)** The biosynthesis pathway of DHAA derived from ethanol constructed in *S. cerevisiae*. All over-expressed genes are marked in red. The genes with star superscripted are over-expressed in multi-copy 2 μ plasmid pRS425 and others are over-expressed in the genome with strong promoter. For gene tHMG1, it is over-expressed with three copies in the genome, which is labeled as ‘x3.’ **(B)** Liquid chromatograms results for the mix standard of DHAA and AA (line in yellow), and the initial strain for DHAA production (line in blue). The DHAA eluted at about 6.67 min and AA eluted at 7.43 min. **(C)** The production of DHAA and AA in strain Sc085. And the ratio of DHAA/AA were shown below.

A 10 μL aliquot was injected into waters e2695 HPLC with ultraviolet detection at 194 nm. A thermoHypersil BDS C18 column (4.6 mm × 150 mm × 5 μm) was used for separation, with the following gradient (channel A: acetonitrile, channel B: water plus 0.1% formic acid): 0–3 min 65% A, gradually increased to 100% A from 3 to 10 min, held at 100% A from 10 to 13 min, decreased to 65% A from 13 to 15 min, kept 65% A from 15 to 18 min. The column was held at 25°C during the separation. Under this condition, DHAA and AA were found to elute at 6.67 and 7.43 min, respectively (see Figure 2B). The concentrations of both products in the sample were calculated using the calibration curves of standards (HPLC ≥ 98%) which was purchased from Chengdu Pufei De Biotech, Co., Ltd. Standards of DHAO, AO, AOH, and AD were purchased from TRC (Toronto Research Chemicals).

### Homology Modeling, Molecular Docking, and Structural Analysis

Three-dimensional structure models of ALDH1 were constructed using the program Swiss-Model^2^ to get the structure information accordingly. The structures were modeled using the high sequence homology (>41% identified) and high resolution crystal structure of the aldehyde dehydrogenase family protein (indole-3-acetaldehyde dehydrogenase) combined with NAD^+^ from *tomato* as the template (pdb id:5iuw-A). The structure models were subjected to energy minimization using the Swiss-Pdb Viewer. Afterward the docking of enzyme and ligand were performed using the AutoDockVina program (Oleg and Olson, 2010). The docking studies were run with DHAO or AO as ligands and the structure model of ALDH1 with NAD^+^. The DHAO and AO structure files were retrieved from ZINC site (Irwin et al., 2012). The docking cluster analysis was performed in the AutoDockVina program environment, and clusters were characterized by binding energy (in kilocalories per mole). Establishment of dative bonds between ligand, NAD^+^ and the corresponding amino acids were followed by energy minimization. The built complex structural analysis was done using Pymol software (Delano, 2010). The mutation at the specific amino acid site was also introduced using this software, which allowed exploration of the spatial and molecular interactions among amino acids.

## Results and Discussion

### Strain Construction for DHAA Production

Referring to previous work (Westfall et al., 2012), CEN.PK2.1C was selected as the host strain for DHAA production. In order to decouple cell growth and product production, inducible promotor GAL (P*_*GAL*__1_*, P*_*GAL*__7_*, P*_*GAL*__10_*) was explored to control all the overexpressed genes (Peng et al., 2017). *GAL80* was deleted to eliminate the demand of galactose for de-repressing the promoter GAL. To increase the metabolic flux toward FPP, all the genes of MVA pathway (Westfall et al., 2012) were overexpressed in the genome. Among these genes, tHMG1 was integrated into the genome with three copies (Supplementary Figure S1). Three key heterologous genes for DHAA biosynthesis including *ADS, CYP71AV1* and *DBR2* were constructed in a multi-copy plasmid pRS425 for higher level expression. Meanwhile, four other heterologous genes, including *ADH1*, *ALDH1*, *CYB5* and *CPR1*, were integrated in the genome at one copy to enhance the metabolic flux to DHAA (Figure 2A). The strain for DHAA production (Sc085) was characterized in shake flask fermentation and the products were detected by HPLC at a wavelength of 194 nm. The results showed the presence of DHAA and AA, indicated by the observation that the retention time of DHAA and AA peaks was same as the mix standard (6.67 min for DHAA, 7.43 min for AA) (Figure 2B). And 327 mg/L of DHAA and 129 mg/L of AA were successfully detected. The DHAA/AA ratio of 2.53 was much higher than the reported highest ratio (DHAA/AA = 1.67) (Figure 2C) (Chen et al., 2017).

**FIGURE 3:**
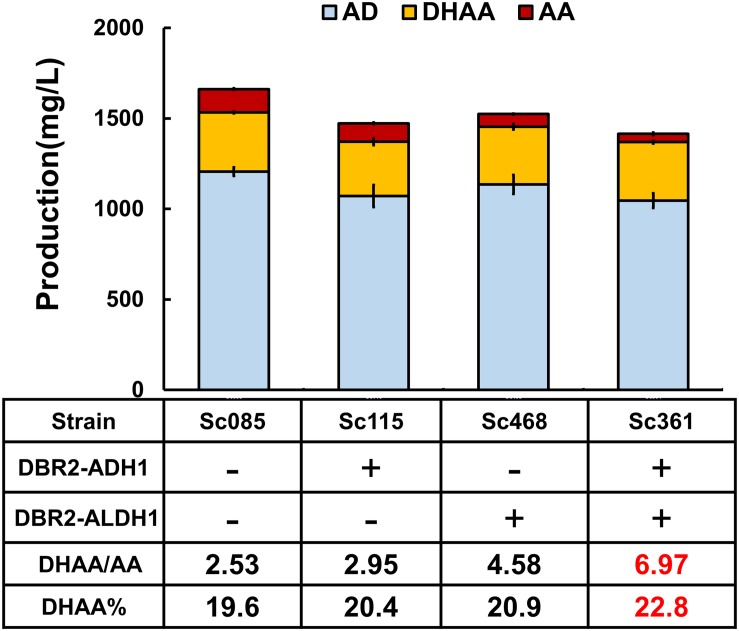
The effects of protein fusions on production of DHAA. DBR2 is individually over-expressed both in pRS425 and genome in strain Sc470, which can be taken as the control to exclude the effect of overexpression of DBR2 on the production of DHAA. Proportion of DHAA (DHAA%) was calculated as the production of DHAA versus the sum production of DHAA, AA, AD, AO, and DHAO.

Nevertheless, there was still about 30% of the metabolic flux flowing toward AA (129 mg/L), probably indicating increasing expression level of DBR2 was needed to enhance the transformation from AO to DHAO. However, because of the reportedly slightly higher affinity of ALDH1 to the joint intermediate AO [Km(ALDH1) = 2.58 μM vs. Km(DBR2) = 19 μM] (Zhang et al., 2008; Teoh et al., 2009), individual over-expression of *DBR2* might not be sufficient to tackle this issue. When Sc470 held another copy of *DBR2* within the chromosome, the DHAA/AA ratio was reduced as well as the DHAA titer and no AOH, AO or DHAO was accumulated (Figure 3 and Supplementary Figure S4). To summarize, it was difficult to directly control the reaction route toward DHAA rather than AA by simply increasing the expression of these genes. Considering that ALDH1 is a promiscuous enzyme that can catalyze AO and DHAO simultaneously, we need to reconfigure the enzymes (ADH1, DBR2, and ALDH1) in a desired order and switch the catalysis preference of ALDH1 to tailor the ratio of DHAA/AA.

### Switch of the Biosynthesis Pathway to DHAA by Fusion Proteins

To increase the substrate accessibility of AO by DBR2, the biosynthetic route from AOH to DHAO was re-built *via* protein fusion of ADH1 and DBR2 (Figure 1). As known, linker type (rigid or flexible) (Lu and Feng, 2008; Chen et al., 2012) and fusion orientation (forward or reverse) (Zhang et al., 2017) could significantly affect the performance of fusion proteins. Borneman and colleagues (Lee et al., 2016) once pointed out that fusing a coumarate-CoA ligase (4CL) with benzalacetone synthase from *Rheum palmatum* (RpBAS) in the 4CL-RpBAS orientation gave rise to significant improvement on final raspberry ketone levels, but the reverse version (RpBAS-4CL) did not work. Therefore, forward and reverse fusions of ADH1 and DBR2 with rigid and flexible linkers (Supplementary Table S2) were constructed and the combined effects on the DHAA output was investigated. As shown in Supplementary Figure S2, it was observed that reverse fusion of ADH1 and DBR2 could significantly increase the ratio of DHAA to AA. To be noted, reverse fusion of ADH1 and DBR2 with rigid linker achieved the highest ratio of DHAA to AA at 2.95, obtaining strain Sc115 (Figure 3 and Supplementary Figure S2). While forward fused protein ADH1-DBR2 tended to accumulate more AA than that of reverse fused protein. To further adjust the ratio of DHAA to AA, increasing the substrate accessibility of DHAO by ALDH1 should also be considered. Based on the result from the fusion of ADH1 and DBR2, the N-terminal of DBR2 should be exposed (Supplementary Figure S2). Thus, the fusion direction here was set as DBR2-ALDH1. Rigid and flexible linkers were also chosen to construct the fusion protein. Strains harboring the fusion protein DBR2-ALDH1 obtained much higher ratio of DHAA to AA compared that in control strain (Sc085) (Supplementary Figure S3). It was also shown that the fusion protein with a rigid linker got the highest ratio of DHAA to AA at 4.58 (Figure 3 and Supplementary Figure S3). Consequently, in order to promote flux from AOH to DHAA, the fusion proteins DBR2-ADH1 and DBR2-ALDH1 were co-expressed to form the strain Sc361 which resulted in a lower production of AA with no concomitant increase in DHAA (323.2 mg/L) and raised the DHAA/AA to 6.76 (Figure 3).

Since there is a high accumulation of AD (about 1.05–1.20 g/L) and no accumulation of any other intermediates (including AO, DHAO, AOH) in all strains described above (Figure 3 and Supplementary Figures S2, S3), the oxidation of AD was also the final step of biosynthesis pathway for all strains, and AOH could be converted to the end product AA or DHAA in time. Additionally, if comparing the effect of the fusion proteins DBR2-ADH1 and DBR2-ALDH1 on the DHAA/AA ratio, DBR2-ALDH1 exhibited a more important role in tailoring DHAA/AA ratio (from 2.53 to 4.58), while the other only increased DHAA/AA ratio to 2.95. Although the sum of the product was reduced, combining two fusion proteins would slightly increase the conversion yield of DHAA transformed from AD [proportion of DHAA (DHAA%) increased from 19.7 to 22.8%]. All of the above data demonstrated that engineering minimal conversion of AO to AA is crucial to increase the DHAA/AA ratio and slightly increase the DHAA%. Thus, in order to get optimal ratio of DHAA to AA, reducing the catalysis preference of the shared enzyme ALDH1 to AO or enhancing the catalysis preference to that of DHAO to smooth flux toward DHAA seems to be especially important, in addition to protein fusion.

### Improving the Ratio of DHAA/AA by Changing ALDH1 Specificity

ALDH1 can employ AO or DHAO as the substrate and may have a slightly stronger binding affinity to AO, which will potentially restrain the production of DHAA (Supplementary Table S1). Herein, we attempted to change the substrate specificity of ALDH1 by rationally modifying its structure to improve the preference to DHAO.

Resorting to the molecular structure, DHAO and AO structures are very similar. They both have the aldehyde group which will then be oxidized to the carboxyl group. Nevertheless, the AO molecule has an additional vinyl group adjacent to the aldehyde group (Supplementary Figure S5) that forms a conjugated structure. Such conjugated structure in AO will affect the electronic cloud distribution of the catalyzed aldehyde group. Therefore, the structure difference of the catalyzed site of substrates would cause distinct binding force of the shared ALDH1 to DHAO or to AO. By analyzing the complex structure of ALDH1 with its cofactor NAD^+^ and substrates, it was found that the nicotinamide group of NAD^+^ just bound to the catalytic site of substrates in the active pocket of ALDH1 (Supplementary Figure S5). Consequently, a hypothesis was proposed that the binding characteristic of NAD^+^ to DHAO or to AO potentially made the crucial contributions to the differences in ALDH1’s affinities to its substrates. With this regard, the structural modifications of ALDH1 were carried out in two aspects. Firstly, the binding pocket, which for both NAD^+^ and substrate, was downsized to shorten the distance between the nicotinamide group of NAD^+^ and the catalyzed aldehyde group of DHAO. Secondly, the binding force of ALDH1 and NAD^+^ was enhanced to improve the complex structural stability and enhance the enzymatic catalytic activity to DHAO, since NAD^+^ is loosely bound to ALDH1 (Butterworth, 2010).

Accordingly, an attempt was made to replace the residues G223 and G227 with branch chains of a larger size (like V or F) by squeezing them through the space within the binding pocket. However, by further comparing the complex structures between the wild type ALDH1/NAD^+^ and mutant (G223V/F, G227V/F)/NAD^+^, it was found that although the binding pocket structure of the mutant complex was downsized, the adenine group of NAD^+^ was extruded out of the original position, due to the steric effect of large branch chains of the mutated residues (Supplementary Figure S6). This structure weakened the binding force between NAD^+^ and ALDH1 mutants (Supplementary Figure S6), probably damaging or even destroying the enzymatic activity of ALDH1 mutants. As expected, either the ratio of DHAA/AA or the DHAA titer were dramatically decreased by mutagenesis of G223F and G227V/F (Supplementary Figure S6F), compared to that of the wild type ALDH1. Although G223V increased the ratio of DHAA/AA, the actual production of DHAA was reduced significantly (Supplementary Figure S6F). The significant accumulation of DHAO was found in all mutants at G223 and G227, showing that the activity of these ALDH1 mutants were reduced. Although CYP71AV1 was reported to transform AO to AA (Teoh et al., 2009), its activity might not be strong enough to produce more AA or DHAA.

**FIGURE 4:**
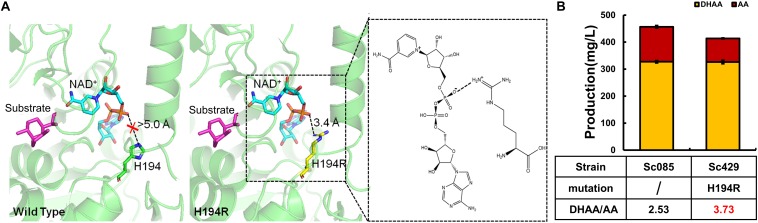
Protein engineering of ALDH1 with mutation H194R and its effect on production of DHAA. **(A)** The complex three-dimensional structures of (left) wild type ALDH1/NAD^+^ and (right) mutant H194R/NAD^+^ with DHAO. Substrate DHAO and cofactor NAD^+^ are colored in purple and cyan, respectively. The electrostatic interaction is boxed with a dotted line. **(B)** The effect of H194R on DHAA production.

Alternatively, we sought to enhance the binding between ALDH1, NAD^+^ and the substrate. The site-directed mutant H194R was constructed to enhance the binding of enzyme to NAD^+^. Comparing with the complex structures of wild type ALDH1/NAD^+^, the mutant H194R formed an additional electrostatic interaction with the phosphate group of NAD^+^ (Figure 4A). Such electrostatic interactions would improve the structural stability of the mutant complex, which would significantly regulate the enzymatic catalysis activity to substrate (AO and DHAO).

Consequently, H194R significantly increased the ratio of DHAA/AA to 3.73 without reducing the production of DHAA (Figure 4). Different from other mutants in G223 or G227, the strain with mutant H194R (Sc429) didn’t accumulate DHAO and any other intermediates (AO, AOH) in the product. H194R seemed to affect the activity of ALDH1 with AO and reduced the production of AA (see Supplementary Figure S6F). In summary, our experimental results were well-consistent with our modeling based on structural analysis (Figure 4).

### DHAA Enrichment by 5 L Fed-Batch Fermentation

**FIGURE 5:**
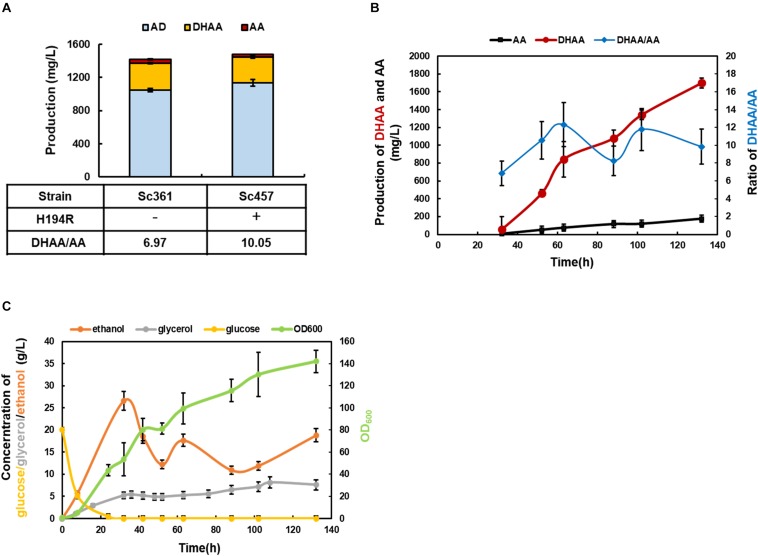
The characterization of the final strain Sc457 and related bioprocess development in 5 L bioreactor. **(A)** The DHAA production in strain Sc457 in which mutated ALDH1(H194R) was introduced into strain Sc361 to replace the wild type of ALDH1 existing in fusion protein DBR2-ALDH1. **(B)** The DHAA production and DHAA/AA ratio of Sc457 in 5 L bioreactor fermentation during the time course. **(C)** The concentrations of major metabolites (glucose, ethanol, glycerol, acetate) during the fermentation.

Mutated ALDH1(H194R) was introduced into strain Sc361 to replace the wild type of ALDH1 existing in fusion protein DBR2-ALDH1, obtaining strain Sc457. As shown in Figure 5A, by further reducing the production of AA, the highest ratio of DHAA/AA at 10.05 was achieved at shake flask level. The DHAA titer was maintained at 311 mg/L without accumulation of AOH, AO, or DHAO (Figure 5 and Supplementary Figure S4). Taking the DHAA/AA ratio and the DHAA titer together into considerations, strain Sc457 was chosen for the following fermentation process.

In order to promote DHAA production and investigate the performance of strain Sc457 in fermenters, 5 L bioreactor fermentation was conducted using the carbon restriction strategy. As shown in Figure 5B, OD_600_ increased sharply before 32 h when glucose was utilized as a carbon source. When the carbon source was switched to ethanol, the OD_600_ was gradually promoted to 142 until harvest. Under above control process, the highest reported DHAA titer at 1.70 g/L in microbes was accomplished after 132 h fermentation (Figure 5B). A slight accumulation of AA was also obtained. It was also observed that the average ratio of DHAA to AA during the fermentation process was quite steady, demonstrating that the genetic stability of strain Sc457 was quite stable and the process was scalable to mimic the strain performance in shake flask to some extent. As the current DHAA production is not as high as that of AA in Amyris, Inc. (Paddon et al., 2013), further optimization of the fermentation efficiency via integrating medium and feed strategy (Westfall et al., 2012) as well as off-gas analysis feedback control could be tried.

During the fermentation process, a significant accumulation of glycerol was also found at about 5 g/L in glucose consumption stage. Ho and colleagues reported both UBR2 and GUT1 with single point mutation could be regarded as targets for establishing glycerol utilization in strains of the CEN.PK family (Ho et al., 2017). It was also found that regulatory and metabolic trade-offs of glycerol utilization in *S. cerevisiae* were revealed by laboratory evolution, which could guide us to enhance glycerol consumption rationally in our study (Strucko et al., 2018). Furthermore, no acetate was detected during the whole process due to carbon restriction strategy (data not shown).

## Conclusion

The combination of metabolic engineering and protein engineering has shown great performances in constituting microbial cell factories for the production of natural products with complex structures. In this study, fusion proteins and modifying ALDH1 catalysis preference strategies have been adopted and conducted to successfully tailor the ratio of DHAA/AA in *S. cerevisiae*. Promoted flux toward DHAA *via* ADH1, DBR2 and ALDH1 was firstly reconstituted by pairing fusion proteins DBR2-ADH1 and DBR2-ALDH1. The theoretical model of enhancing the stability of the enzyme/cofactor complex was assumed and executed to switch the catalysis preference of ALDH1 toward DHAA. Consequently, the ratio of DHAA/AA was elevated from 2.53 to 10.05 with the highest DHAA titer reaching 1.70 g/L (DHAA/AA ratio of 9.84) in 5 L bioreactor fermentation. This study shows the potential of oriented arrangement of consecutive heterologous enzymes in the reconstitution of microbial cell factories. On the other hand, the reduced sum flux of the biosynthesis pathway might be enhanced by other strategies of metabolic engineering which will be studied in future work.

## Data Availability Statement

The datasets generated for this study can be found in the ADS (ACCESSION Q9AR04), CYP71AV1 (ACCESSION Q1PS23), DBR2 (ACCESSION KC505370.1), ALDH1 (ACCESSION JQ609276.1), ADH1 (ACCESSION JF910157.1), CYB5 (ACCESSION JQ582841.1), and CPR1 (ACCESSION DQ318192.1).

## Author Contributions

B-XZ and W-HX conceived of the study and participated in fed-batch fermentation. B-XZ and YW participated in strain construction and carried out the molecular genetic studies. M-DY carried out the protein analysis. M-DY, YW, and W-HX participated in design and coordination of the study as well as helped to draft the manuscript. W-HX supervised the whole research and revised the manuscript. All the authors read and approved the final manuscript.

## Conflict of Interest

The authors declare that the research was conducted in the absence of any commercial or financial relationships that could be construed as a potential conflict of interest.

## Footnotes

1 http://parts.igem.org/Part:pSB1C3

2 http://swissmodel.expasy.org/
