# Supplementary material for: Metabolic Engineering of Saccharomyces cerevisiae for Enhanced Dihydroartemisinic Acid Production
Source: Front Bioeng Biotechnol. 2020 Mar 17;8:152. doi: 10.3389/fbioe.2020.00152 (PMC7090239; doi:10.3389/fbioe.2020.00152)
Supplement: Supplementary file 1 [file Table_1.DOCX]

Supplementary Material

1. **Supplementary Tables**

**Table S1.** The enzymatic parameter of DBR2 and ALDH1

|  | Km (μM) | | Kcat (s^-1^) | | Kcat/Km (s^-1^·μM^-1^) | | reference |
| --- | --- | --- | --- | --- | --- | --- | --- |
|  | AO | DHAO | AO | DHAO | AO | DHAO |  |
| DBR2 | 19 | / | 2.6 | / | 0.14 | / | (Yansheng et al., 2008) |
| ALDH1 | 2.58 | 8.79 | 1.53 | 7.74 | 0.59 | 0.88 | (Teoh et al., 2009) |

**Table S2.** Primer used in this study

| **Primer** | **Sequence** |
| --- | --- |
| 2u-ADH1t-F | gataagcttgatatcgaattcctgcagCATGCCGGTAGAGGTGTGGTC |
| 2u-ADH1t-R | TGTTGGTTCCATCTTTCTAAAGCGAATTTCTTATGATTTATGATTT |
| 2u-ADS-F | TTTTTGAAAATTCAATATAAATGTCTTTGACTGAAGAAAAGCCA |
| 2u-ADS-R | CGATTTCAATTCAATTCAATTTAGATAGACATTGGGTAAACCAACAAAG |
| 2u-Pgal1,10-F | AGCCTTCAAGATAGACTTCATTATAGTTTTTTCTCCTTGACGTTAAAG |
| 2u-Pgal1,10-R | CTTTTCTTCAGTCAAAGACATTTATATTGAATTTTCAAAAATTCTTAC |
| 2u-CYP71AV1-F | AAATCATAAGAAATTCGCTTTAGAAAGATGGAACCAACAACAATTC |
| 2u-CYP71AV1-R | GTCAAGGAGAAAAAACTATAATGAAGTCTATCTTGAAGGCTATGGCT |
| 2u-PGK1t-F | GTTTACCCAATGTCTATCTAAATTGAATTGAATTGAAATCGATAGATC |
| 2u-PGK1t-R | CTAGTGGATCCCCCGGGCTGCAACGAACGCAGAATTTTCGAGTTA |
| DCA-Pgal7-F | AATCCCCGCTCGAGGATTTGCCAGCTTACTATCCTTCTTG |
| DCA-Pgal7-R | CAAGGTTGGCTTTTCAGACATTTTTGAGGGAATATTCAACTGTTTTTTTTTATC |
| DCA-DBR2-F | AAAAACAGTTGAATATTCCCTCAAAAATGTCTGAAAAGCCAACCTTGTTCTC |
| DCA-DBR2-R | CAAAGGAAAAGGGGCCTGTTTACAACAAAGAACCTTGGTCCAAAG |
| DCA-CYC1t-F | AGGTTCTTTGTTGTAAACAGGCCCCTTTTCCTTTGTCG |
| DCA-CYC1t-R | CATTGGTTCTGCAGGTCGACAACTAAACTGGAATGTGAG |
| AD-pGAL7-FN | AAggAAAAAgCggCCgCgATTTgCCAgCTTACTATCCTTCTTgA |
| DA-cyc1t-F | gTgTTAAgATCTTgATCAAgTTCTAAACAggCCCCTTTTCCTTTgTCg |
| DA-cyc1t-R | TTTTTCgCggATCCCATTggTTCTgCAggTCgACAACTAAACTggAATgTgAg |
| AD-DBR2-F1 | gCAAgCAgAAgCCgCTgCAAAAgAAgCggCTgCTAAAgCTTCTgAAAAgCCAACCTTgTTCTCTgCTTAC |
| AD-ADH1-R1 | TTTAgCAgCCgCTTCTTTTgCAgCggCTTCTgCTTgCAAgAACTTgATCAAgATCTTAACACAgTCTgg |
| AD-ADH1-R2 | CCggTgCCggAgCAggAgCTggggCAggTgCTggAgCgAACTTgATCAAgATCTTAACACAgTCTgg |
| AD-DBR2-F2 | CTgCCCCAgCTCCTgCTCCggCACCggCTCCAgCTCCATCTgAAAAgCCAACCTTgTTCTCTgCTTAC |
| AD-ADH1-R3 | CgCCACCTCCACTTCCCCCACCgCCTgAACCACCACCTCCgAACTTgATCAAgATCTTAACACAgTCTgg |
| AD-DBR2-F3 | ggTggTTCAggCggTgggggAAgTggAggTggCggTTCTTCTgAAAAgCCAACCTTgTTCTCTgCTTAC |
| DA-DBR2-R1 | AgCTTTAgCAgCCgCTTCTTTTgCAgCggCTTCTgCTTgCAACAACAAAgAACCTTggTCCAAAgATg |
| DA-ADH1-F1 | gCAgAAgCCgCTgCAAAAgAAgCggCTgCTAAAgCTgCTCAAAAggCTCCAggTgTTATCACTTg |
| DA-ADH1-R | CAAAggAAAAggggCCTgTTTAgAACTTgATCAAgATCTTAACACAgTCTgg |
| DA-DBR2-R2 | CCggTgCCggAgCAggAgCTggggCAggTgCTggAgCCAACAAAgAACCTTggTCCAAAgATg |
| DA-ADH1-F2 | CTgCCCCAgCTCCTgCTCCggCACCggCTCCAgCTCCAgCTCAAAAggCTCCAggTgTTATCACTTg |
| DA-DBR2-R3 | CgCCACCTCCACTTCCCCCACCgCCTgAACCACCACCTCCCAACAAAgAACCTTggTCCAAAgATg |
| DA-ADH1-F3 | ggTggTTCAggCggTgggggAAgTggAggTggCggTTCTgCTCAAAAggCTCCAggTgTTATCACTTg |
| 17E0b-hphA-F | TGCGGGATTGCTCTCGGTCAAGCTTGTTTAGCTTGCCTCGTCCCCGCCGGGTCACCC |
| 17E0b-hphA-R | TCATTATCCTCATCAAGATTGCTTTATTGGATGGCGGCGTTAGTATCGAATCGACAGC |
| 17E0-up-R | GGGTGACCCGGCGGGGACGAGGCAAGCTAAACAAGCTTGACCGAGAGCAATCCCGCA |
| 17E0-down-F | GCTGTCGATTCGATACTAACGCCGCCATCCAATAAAGCAATCTTGATGAGGATAATGA |
| 17E0-up-F | TGACAGAGCAGAAAGCCCTAGTAAAGCG |
| 17E0-down-R | CTTTCTTAGTATATATATACTGCTCAAGGGC |
| 17E1-ALDH1-F | ATAATCGCGGATCCTACTAGAGACCCATTCGACTTGGCTACTAGAC |
| 17E1-ALDH1-RP | TTTAATTTCTGCAGTTTAAACTATATATACTGCTCAAGGGCAAATGC |
| 17E1-pGAL7-F | TGCGGTGTGAAATACCGCGATTTGCCAGCTTACTATCCTTCTTG |
| 17E1-His3-FE | ATAACCGGAATTCGTTTAAACGAAGGCAAAGATGACAGAGCAGAAAGC |
| 17E1-His3-R | GATAGTAAGCTGGCAAATCGCGGTATTTCACACCGCATAGA |
| 17E1x-DBR2-R | ATATGGTCTCCCAGGAGCTGGGGCAGGTGCTGGAGCCAACAAAGAACCTTGGTCCAAAG |
| 17E1x-ALDH1-F | AAAGGTCTCACCTGCTCCGGCACCGGCTCCAGCTCCATCTTCTGGTGCTAACGGTTCTT |
| 17E1-ALDH1-R | TCCTTAGCGATAGTCATGTCGTCAGT |
| 17E1y-DBR2-R | AAATCGGTCTCACACCGCCTGAACCACCACCTCCCAACAAAGAACCTTGGTCCAAAG |
| 17E1y-ALDH1-F | AAAGGTCTCAGGTGGGGGAAGTGGAGGTGGCGGTTCTTCTTCTGGTGCTAACGGTTCTT |
| 17E1z-DBR2-R | AAATCTGGTCTCGTCTTTTGCAGCGGCTTCTGCCAACAAAGAACCTTGGTCCAAAG |
| 18E5-FK | TCGCCCAATGGTACCAACGATGTTCCCTCCACCAAAGG |
| 18E5dR-R | AAACAGTCAATGGAGTTCTTTCAGCTGGCTTGATAACCATAGTAC |
| 18E5dR-F | CAGCTGAAAGAACTCCATTGACTGTTTTGTTCTTG |
| 18E5aF-R | GCAGTCTTAAAGAAACCGTTAACAACGTTGATAAC |
| 18E5aV-R | GCAGTCTTAACGAAACCGTTAACAACGTTGATAAC |
| 18E5aF-F | TCAACGTTGTTAACGGTTTCTTTAAGACTGCTGGTGCTGCTGTTTC |
| 18E5aV-F | TCAACGTTGTTAACGGTTTCGTTAAGACTGCTGGTGCTGCTGTTTC |
| 18E5bF-R | GAGAAGAAACAGCAGCAAAAGCAGTCTTACCGAAACCGTTAAC |
| 18E5bV-R | GAGAAGAAACAGCAGCACAAGCAGTCTTACCGAAACCGTTAAC |
| 18E5bF-F | AGACTGCTTTTGCTGCTGTTTCTTCTCACATGGAC |
| 18E5bV-F | AGACTGCTGTTGCTGCTGTTTCTTCTCACATGGAC |
| 18E5cF-R | ATAACAGTTCTACCAAATTCAGTAGAACCAGTGAAAGTAACC |

1. **Supplementary Figures**

**Figure S1.** Schematic map of over-expression of genes in the DHAA biosynthesis pathway for construction of original strain Sc085


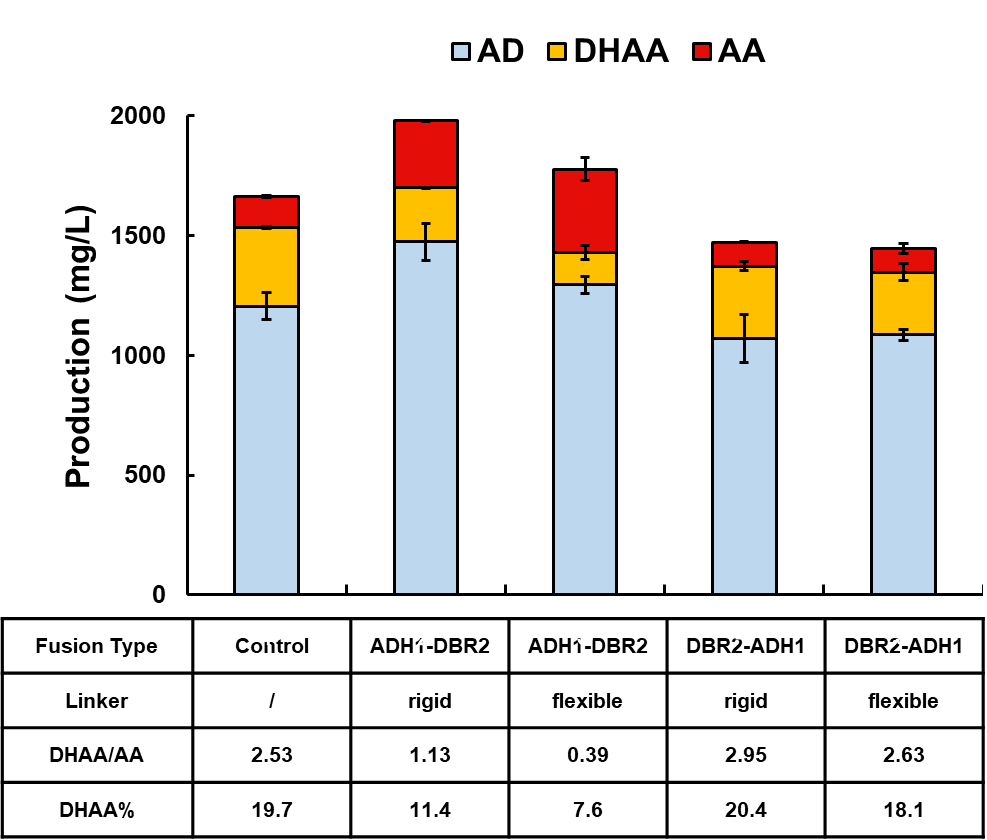


**Figure S2.** The combinatorial effects of fusion direction and linker type of fused protein ADH1-DBR2 on production of DHAA.


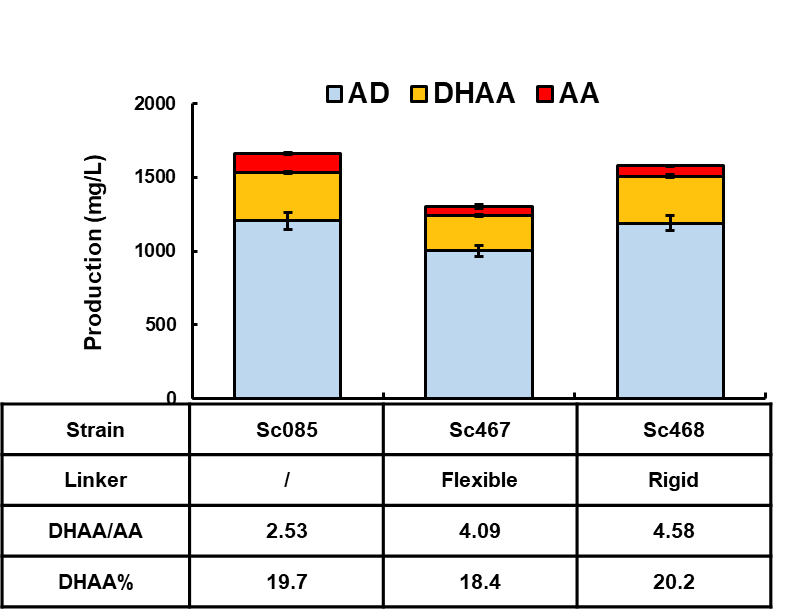


**Figure S3.** The effect of linker type of fused protein DBR2-ALDH1 on the production of DHAA


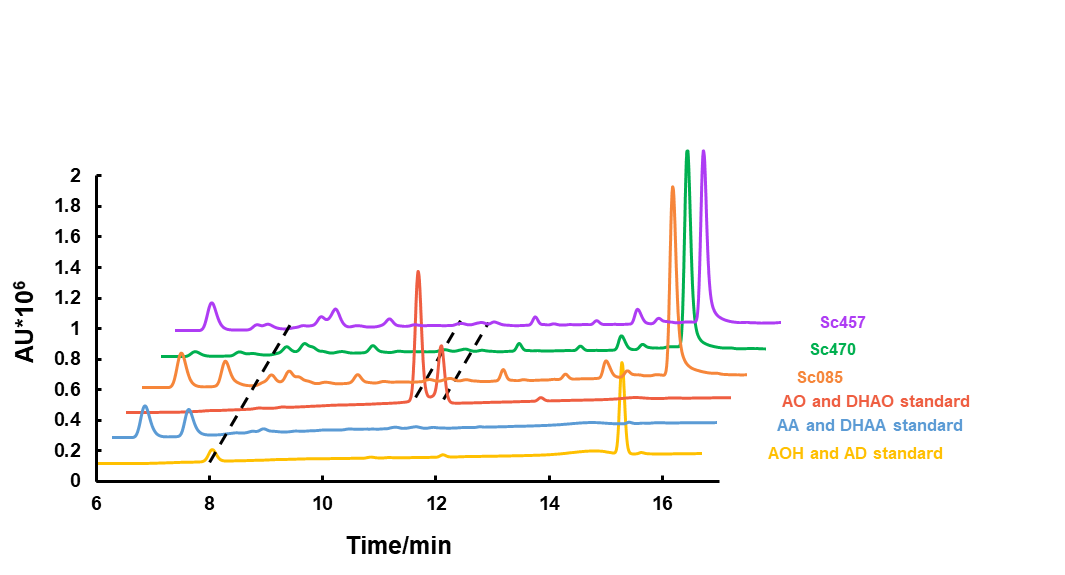


**Figure S4.** HPLC result for production of strain Sc085, Sc470, Sc457 and the standard of intermediates mixture: AOH and AD in yellow; AA and DHAA in blue; AO and DHAO in red. DHAA, AA, AOH, AO, DHAO and AD eluted at 6.67min, 7.43min, 8.72min, 11.35min, 11.70min, and 14.82min. The dash line shows no accumulation of AOH, AO, DHAO in final strain Sc457 and original strain Sc085


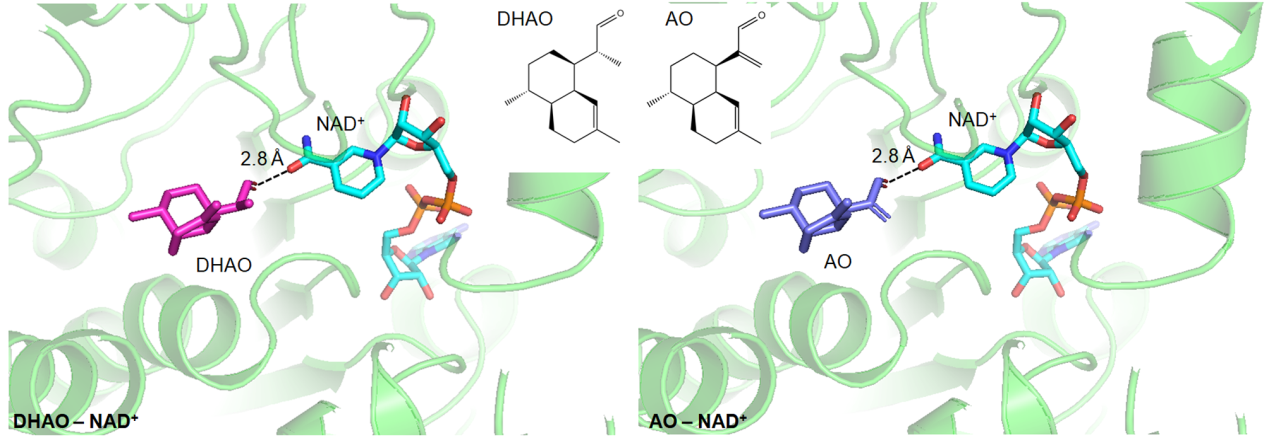


**Figure S5.** The three-dimensional structures of the complex of ALDH1/NAD^+^ with DHAO (left) or AO (right). The molecule structures of DHAO and AO were indicated.


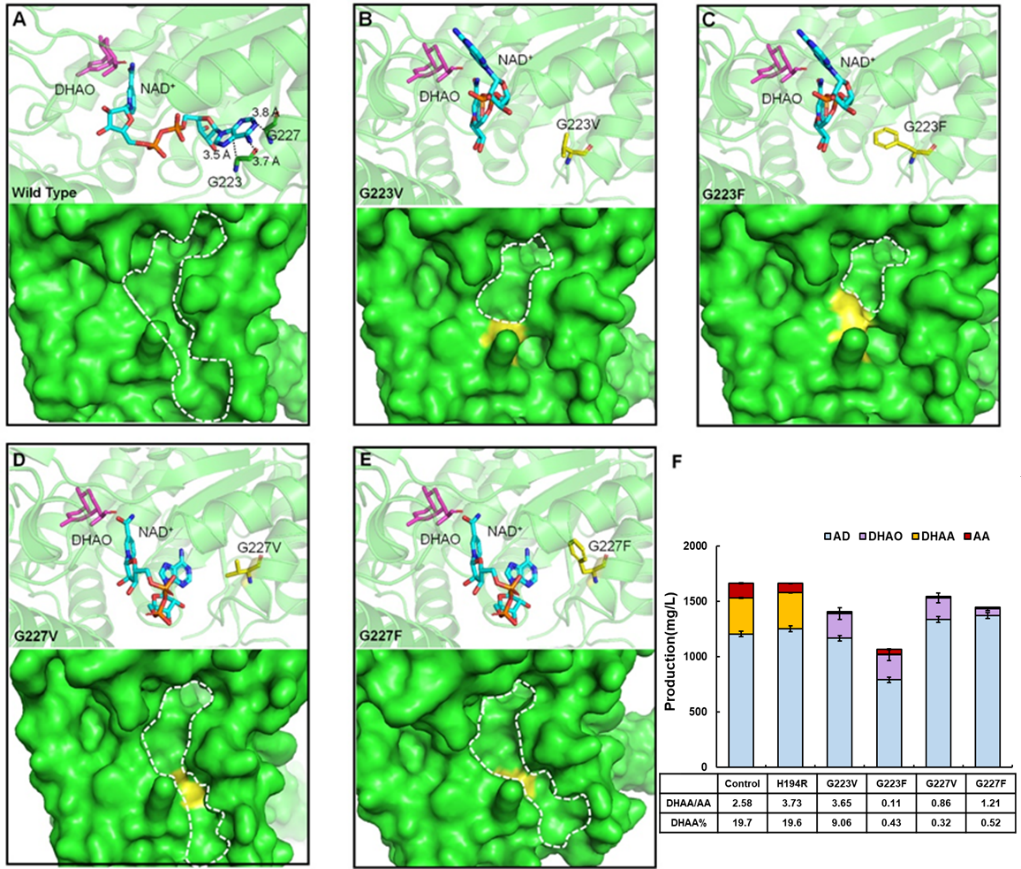


**Figure S6.** The complex three-dimensional structures (top) and surface structures (down) of (A) wild type ALDH1/NAD^+^/DHAO and (B-E) mutant (G223V/F, G227V/F)/NAD^+^/DHAO. The activity pockets were boxed in white dotted lines. The mutated residues were labeled in yellow (F) The effect of G223V/F, G227V/F, H194R on DHAA, DHAO, AA and AD production

**Reference**

Teoh, K. H., Polichuk, D. R., Reed, D. W., Covello, P. S., 2009. Molecular cloning of an aldehyde dehydrogenase implicated in artemisinin biosynthesis in Artemisia annua. Botany-botanique. 87, 635-642.

Yansheng, Z., Teoh, K. H., Reed, D. W., Lies, M., Alain, G., Olson, D. J. H., Ross, A. R. S., Covello, P. S., 2008. The molecular cloning of artemisinic aldehyde Delta11(13) reductase and its role in glandular trichome-dependent biosynthesis of artemisinin in Artemisia annua. Journal of Biological Chemistry. 283, 21501.
